# Evaluating cytotoxicity of methyl benzoate *in vitro*

**DOI:** 10.1016/j.heliyon.2020.e03351

**Published:** 2020-02-04

**Authors:** Heeyoun Bunch, Jungeun Park, Hyeseung Choe, Md Munir Mostafiz, Jang-Eok Kim, Kyeong-Yeoll Lee

**Affiliations:** a School of Applied Biosciences, College of Agriculture and Life Sciences, Kyungpook National University, Daegu 41566, Republic of Korea; b Institute of Agricultural Science and Technology, Kyungpook National University, Daegu 41566, Republic of Korea

**Keywords:** Agricultural science, Pesticide, Environmental health, Toxicology, Methyl benzoate, Cytotoxicity, Gene expression

## Abstract

Methyl benzoate (MB) is a small, hydrophobic organic compound that is isolated from the freshwater fern, *Salvinia molesta*. Because of its pleasant odor, it has been used as a fragrance and flavor enhancer. In addition, it is used to attract orchid bees for pollination in the farm and has been tested for its potential to be developed as a green pesticide targeting a diverse group of insects. In spite of its wide applications, the safety of MB to humans remains poorly understood. In this study, we tested the cytotoxicity of MB against cultured human cells, including kidney, colon, and neuronal cells. Furthermore, other natural and synthetic benzoic acids such as ethyl benzoate (EB) and vinyl benzoate (VB) were compared with MB for their similarity and broad commercial and industrial applications. We found that MB and VB have the least and most overall toxicity to the tested human cells, respectively. In addition, the expression of some genes involved in cell cycle, protein quality control, and neurotransmission such as *cyclin D1*, *HSP70*, and *ACHE* genes was differentially expressed in the presence of these chemicals, most noticeably in treatment of VB. Our study provided the LC_50_ values of these benzoic acids for human cells *in vitro* and suggested their mild toxicity that should be considered in the industrial and agricultural applications to be within safe limits.

## 1 Introduction

Sustainable agriculture fits into modern agriculture and aims to promote farming practices and methods that are profitable, environment-friendly, and good for communities. The growing population demands an increase in the amount of agricultural produces, either by expanding the farming areas or intensification of agriculture (Pretty and Bharucha, 2014). The latter is more ideal because farming area expansion would be achievable only at the expense of environments and habitable lands. To increase the agricultural production per unit area, the usage of pesticides including herbicides and insecticides is unavoidable and has been widely accepted as a typical agricultural practice (https://www.epa.gov/sites/production/files/2017-01/documents/pesticides-industry-sales-usage-2016_0.pdf). Over 95% of pesticides reach other organisms apart from the targeted insects and herbs because they are typically sprayed or spread over the farming area (Feng and Zhang, 2017). Therefore, unless pesticides are carefully monitored for their safety and long-term effects on human health and environment, they can cause irreversible harms and disastrous outcomes to them. In fact, some synthetic pesticides have reportedly harmed and posed risks to human health and environments in the past (Aktar et al., 2009). It is worthy to note that many insect pests have acquired resistance to the pesticides that are used currently, demanding the development of the new pesticides (Georghiou and Mellon, 1983). Depending on their origin, pesticides are categorized as natural, naturally derived, or synthetic. Synthetic pesticides are recognized as more hazardous than natural or naturally derived ones. However, natural pesticides can be carcinogenic and as damaging as synthetic pesticides.

Methyl benzoate [MB; C_8_H_8_O_2;_ molecular weight (MW), 136.15 g/mol] is a volatile ester that exists naturally as a metabolite in plants (Dudareva et al., 2000). It was isolated from freshwater fern *Salvinia molesta* for the first time about 10 years ago (Choudhary et al., 2008). It is also the most abundant fragrant compound in bee-pollinated snapdragon flowers (Dudareva et al., 2000). S-Adenosyl-l-methionine: benzoic acid carboxyl methyltransferase (BAMT) is responsible for the biosynthesis of MB in snapdragon flowers (Dudareva et al., 2000). BAMT catalyzes the transfer of the methyl group of S-adenosyl-l-methionine to the carboxyl group of benzoic acid to generate MB (Kolosova et al., 2001). In addition, MB is emitted from insect-damaged rice plants (Zhao et al., 2010). Because of its fruity smell, it is used in perfumery and as a pesticide to attract orchard bees (Breed et al., 2004). Recent studies have demonstrated toxic effects of MB against various insect pests including *D. suzukii*, *H. halys*, and *Bemisia tabaci* (Feng et al., 2018; Feng and Zhang, 2017; Mostafiz et al., 2018). Other natural benzyl esters, such as ethyl benzoate (EB) and synthetic benzoate, such as vinyl benzoate (VB), have been shown to have insecticidal effects on some insect pests (Feng et al., 2018). In this study, given that MB and EB have the great potential to be developed into green pesticides with less impact on the environment (Feng et al., 2018), we examined their cytotoxicity in cultured human cells, including human embryonic kidney (HEK293), colon (CACO2), and neuronal (SH-SY5Y) cells *in vitro*. Also, the effect of these chemicals on cell proliferation was monitored by gene expression levels and the cellular stress response was examined by *HSP70* expression. In our study, we propose to provide the valuable data to help set the safety limits of MB, EB, and VB for human applications and use in pesticides in future.

## 2 Materials and methods

### 2.1 Cytotoxicity tests

Human embryonic kidney 293 (HEK293) and CACO2 cells were purchased from ATCC. Cells were cultured in DMEM (Corning, USA) supplemented with 10% FBS (Gibco, USA) and 1% penicillin/streptomycin (Gibco, USA). SH-SY5Y cells were gifted by Dr. Dong-Hyung Cho's laboratory in the Dept. of Life Science at Kyungpook National University and were grown in DMEM with high glucose (Gibco, USA). Cells were grown to 80–90% confluence in a 10 cm plate before splitting into a 96 well plate. Approximately 4 × 10^4^ cells were seeded in each well and MB, EB, or VB was applied according to targeted concentrations. MB, EB, and VB was dissolved in 100% acetone and the solution was added to the cell media as 2% (v/v, 3 μl MB solution to 150 μl cell culture media). For the experiments with Tween 20, 1% Tween 20, 80% acetone, and 19% H_2_O were used as a solvent to dissolve MB. The MB solution including Tween 20 was added to the cell media as 2% as described above. After 48 h incubation, 10% (v/v) of water soluble tetrazolium salt (WST, DoGen Inc., South Korea) was added to each well, following the manufacturer's instruction. Orange color development for enzyme-substrate reaction was monitored, using spectrophotometry at 450 nm (Tecan Sunrise, Switzerland).

For the crystal violet experiment, cells were split into a 96 well and MB was applied to each well as described above and then HEK293 cells were grown for 1–2 days and CACO2 cells for 4–5 days. Crystal violet staining method published by M. Feoktistova et al., (2016) was used without modifications. The resultant plate was taken picture of. In addition, the stained cells were dissolved in methanol and the intensity of color was measured at 595 nm (Tecan Sunrise, Switzerland).

### 2.2 Real-time PCR

HEK293 and SH-SY5Y cells were grown to 60–70% confluence in 6 well plates and the media were exchanged with the ones including the chemicals, MB, EB, or VB in the given concentrations. After 48 h incubation, the cells were washed with cold PBS once and scraped. The cells were washed again with cold PBS twice before extracting total RNA molecules using Qiagen RNeasy kit. cDNAs were constructed from 600 ng of the collected RNAs using Reverse Transcription System (Promega, Japan). cDNA was analyzed through real-time quantifying PCR using SYBR Green Realtime PCR Master Mix (Toyobo, Japan) according to the manufacturer's instructions (CFX96 Touch Real-time PCR System, Bio-Rad). The primers used for the experiments are summarized in Table 2.

**Table 1:** LC50 values of MB, EB, and VB in human kidney, colon, and neuron cell lines.

Cell	Compound	LC_50_ (%/mM)	95% CI (lower-upper)	Slope ± SEM	χ^2^ (df)
HEK293	MB	0.18/13.2	1.81–245.45	1.24 (0.11)	120.06 (4)
	EB	0.104/6.9	n/a	2.9 (1.5)	38.6 (2)
	VB	0.08/5.4	0.07-0.09	2.4 (0.3)	3.3 (2)
CACO2	MB	0.28/20.6	0.06-1.94	1.07 (0.1)	15.17 (4)
SH-SY5Y	MB	0.15/11.0	n/a	2.0 (1.2)	44.7 (2)
	EB	0.11/7.3	n/a	3.2 (2.5)	90.3 (2)
	VB	0.09/6.1	n/a	4.6 (13.4)	34.6 (2)

CI, confidence interval; the LC50 value was calculated using percentage mortality; n/a, no confidence interval observed and therefor no probit analysis performed.

**Table 2:** Primers used for the real-time PCR.

Target gene	Primer sequence
Cyclin B1	Forward: 5′-AAT GAA ATT CAG GTT GTT GCA GGA G-3′
	Reverse: 5′-CAT GGC AGT GAC ACC AAC CAG-3′
Cyclin D1	Forward: 5′-ATG TTC GTG GCC TCT AAG ATG A-3′
	Reverse: 5′-CAG GTT CCA CTT GAG CTT GTT C-3′
HSP70	Forward: 5′-ATG TCG GTG GTG GGC ATA GA-3′
	Reverse: 5′-CAC AGC GAC GTA GCA GCT CT-3′
ACTIN	Forward: 5′-GCC GAC AGG ATG CAG AAG GAG ATC A-3′
	Reverse: 5′-AAG CAT TTG CGG TGG ACG ATG GA-3′
ACHE	Forward: 5′-GAG AGG ATC TTT GCT CAG CGA C-3′
	Reverse: 5′-GAG AAA GCG ATT CCA GAA GGC-3′

### 2.3 Western blot

HEK293 cells grown in 6 well plates for Western blots and SDS polyacrylamide gel electrophoresis (SDS-PAGE) were washed with cold PBS twice and scraped in RIPA buffer (Cell Signaling, Cat. 9806, USA). Protein concentration in each sample was measured through Bradford assay using Bio-Rad Protein Assay Dye Reagent Concentrate (Bio-Rad #5000006) and spectrophotometry at 595 nm (Tecan Sunrise™ Absorbance Microplate Reader, Switzerland). From the measured protein concentration, a total of 15 μg of proteins per each sample were loaded on 7% SDS-PA gels, blotted onto nitrocellulous membrane, and probed for HSP70 and α-Tubulin using corresponding antibody (HSP70, Santa Cruz sc-32239; α-Tubulin, Santa Cruz sc-8035) in Western blot assay.

### 2.4 Statistical analysis

One-way ANOVA, followed by a post-hoc Tukey's HSD test, was used to determine differences in toxicity, and repellency percentages (*P* < 0.05) (SAS for Window release 6, SAS Institute). All percentage mortality data were corrected using Abbott's formula (Abbott, 1987). Log-probit regression was used to determine lethal median concentration (LC_50_) based on corrected mortality from various MB concentrations (SAS for Window release 6, SAS Institute). Adult repellency percentage was calculated using the formula PR (%) = [(C-T)/(C + T)] × 100 (Liu et al., 2013). All analyses were performed in SAS version 9.4. All the graphs were drawn with SigmaPlot 12.5 (Systat Software, Inc., San Jose, CA, USA).

## 3 Results

### 3.1 Cytotoxicity of MB against human kidney cells *in vitro*

**Figure 1:**
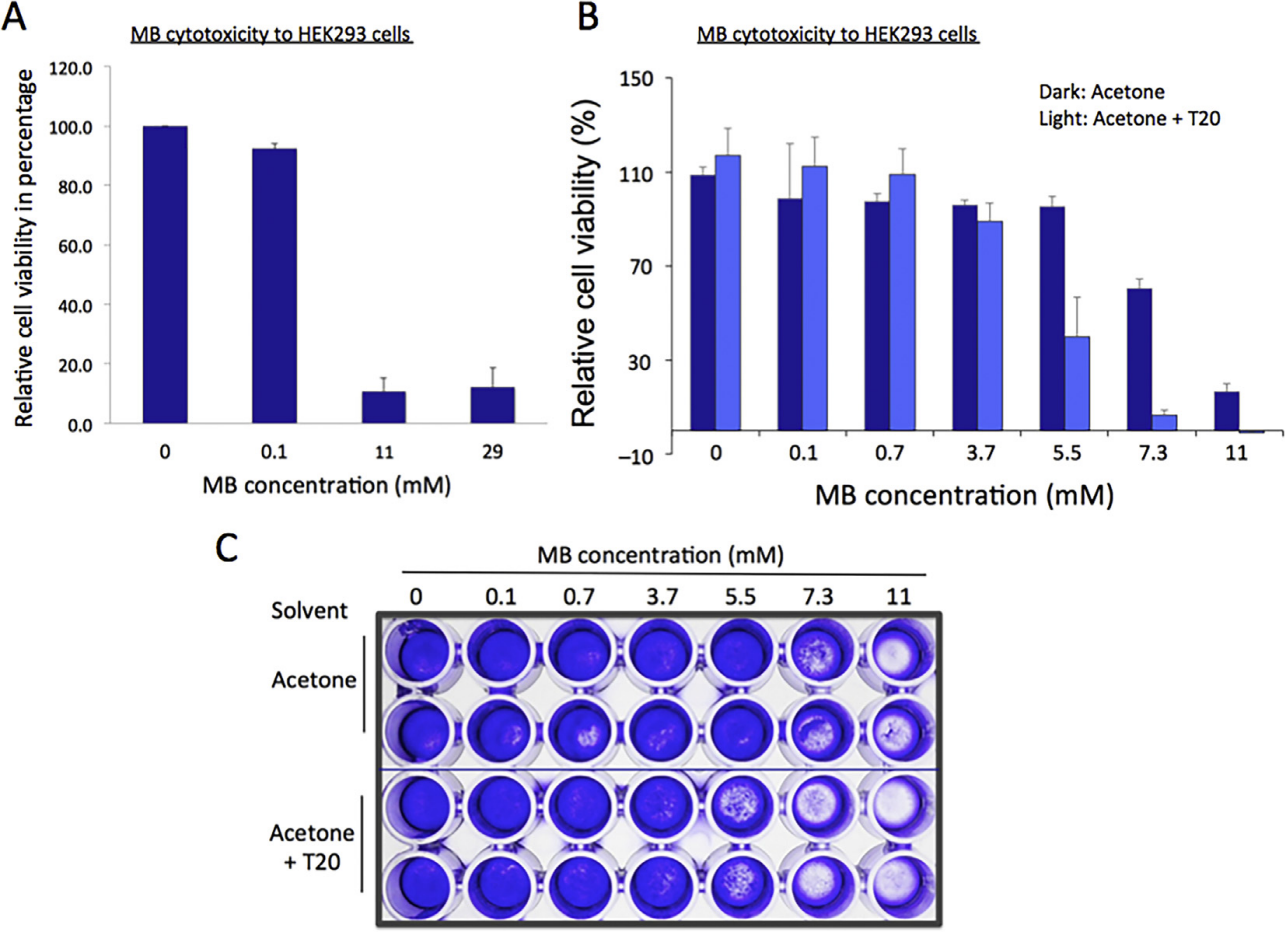
Cytotoxicity of MB against HEK293 cells. Approximately 4 × 10^4^ HEK293 cells were seeded in a well of a 96 well plate along with MB at indicated concentrations. (**A**–**B**) The WST assay results showing the average cell viability relative to the control without MB. Error bars, standard deviations (n = 12 for A; n = 10 for B). Light gray bars, acetone; black ones, acetone with 1% T20 used as a solvent for MB. (**C**) Representative results from the crystal violet assays with HEK293 cells.

As the safety of agricultural products and foods became a major concern and issue in the modern society, validating the effects of pesticides on human physiology and setting the safety limits of their use is important (Fan and Jackson, 1989; Kim et al., 2017). Therefore, we tested MB for its effects on the growth and proliferation of the following: cultured human kidney (HEK293) and colon cells (CACO2) from ATCC. Initially, MB at concentrations of 0, 0.1, 11, and 29 mM (0%, 0.001%, 0.15%, and 0.4%) was applied to approximately 4 × 10^4^ HEK293 cells, and the cells were incubated for 24 h before water-soluble tetrazolium (WST-1) reagent was added. WST-1 is a substrate of mitochondrial dehydrogenases and is converted to a colored dye called formazan in viable cells. Because the intensity of dye is proportional to the amount of enzyme, WST-1 reagent cell proliferation assay is widely used to measure the cell viability (Ngamwongsatit et al., 2008). The results showed that the cells were less viable at >11 mM MB but grew well at 0.1 mM MB when compared with the control (Figure 1A).

Therefore, we aimed to identify an upper limit of MB concentration between 0.1 and 11 mM (0.001% and 0.15%) where the growth of HEK293 cells was less affected. Seven MB concentrations, 0, 0.1, 0.7, 3.7, 5.5, 7.0, 11 mM (0%, 0.001%, 0.01%, 0.05%, 0.075%, 0.1%, and 0.15%), were applied to HEK293 cells and the WST-1 experiment was performed. The results showed that MB concentrations <5.5 mM have little effects on the growth and proliferation of cells, with less than 15% reduction in cell viability as compared with that in the control (Figure 1B). These observations were consistent with those from the crystal violet cell staining assay shown in Figure 1C. It is noted that MB, in concentrations higher than 5.5 mM (0.075%), formed silver-colored aggregates, only visible when applied to the media. This might be because of the hydrophobicity of MB which causes it to form aggregates facilitated by the aqueous condition in the cell culture media. Therefore, 1% Tween 20 (T20) in 80% acetone was used to increase the solubility of MB as a solvent, and the MB mixture was applied to the cell culture at 2% (v/v) (Akbari et al., 2015). The WST-1 data were similar to the results without T20, except for 5.5 mM MB. With the addition of T20, MB concentrations of ≤3.7 mM hardly affected the cell growth and proliferation while MB concentration 5.5 mM inhibited them noticeably (Figure 1B). We attributed the increased sensitivity of MB concentration 5.5 mM (0.075%) in the acetone-T20 solvent to the synergistic cytotoxic effect in the presence of the detergent, T20 with MB or the enhanced solubility of MB. However, the silver aggregate was persistently observed at higher concentrations of MB ≥ 5.5 mM in spite of the addition of T20, suggesting that the former is likely the cause of the increased cell death at 5.5 mM MB. When the cells were visualized with the crystal violet assay, the results showed agreement with those observed in the WST-1 assay, confirming that MB concentrations ≤0.05% could be the upper limit for HEK293 cell viability (Figure 1C).

### 3.2 Cytotoxicity of MB to human colon and neuronal cells *in vitro*

**Figure 2:**
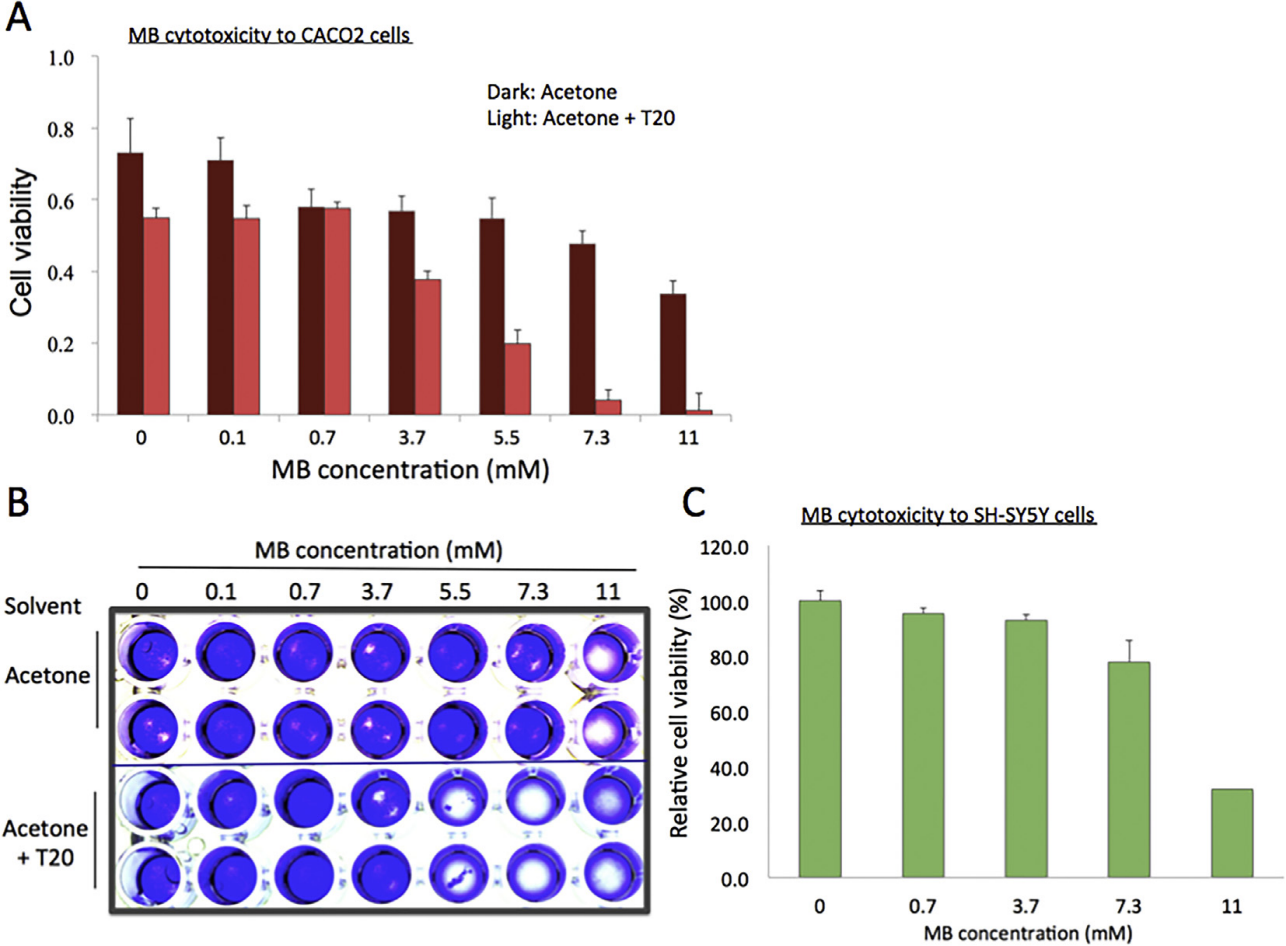
Cytotoxicity of MB against CACO2 and SH-SY5Y cells. (**A**) Results from the WST assays with CACO2 cells. Error bars, standard deviations (n = 6). Light gray bars, acetone; black ones, acetone with 1% T20 used as a solvent for MB. (**B**) Representative results from the crystal violet assays. (**C**) Results from the WST assays with SH-SY5Y cells. Error bars, standard deviation (n = 6).

Ingested residual pesticides from agricultural foods may stay in the colon for a longer time than any other organs. Therefore, MB cytotoxicity to human intestinal epithelial cells, CACO2, was assayed with the WST-1 and crystal violet cell staining assays. Although HEK293 and CACO2 cells have different growth rates, the effects of MB on these two cell lines were similar in both the assays, except that CACO2 cells appeared a bit more resistant to MB. In CACO2 cells, the cell viability reduced <50% at 11 mM MB (approximately 80% reduction in cell viability was observed at this concentration in HEK293 cells) (Figures 1 and 2A). In contrast, increased toxicity toward CACO2 cells was observed for MB dissolved in acetone-T20 (Figure 2A). As shown in Figure 2A, MB concentrations at 3.7 mM (0.05%) and 7.3 mM (0.1%) resulted in about 30% and >90% reduction in CACO2 cells in the WST-1 assay. The results were consistent with the results from the crystal violet assay (Figure 2B).

A compound similar to MB, methyl hydroxylbenzoate or methylparaben is an antifungal agent and serves as a pheromone for a variety of insects (Nathan and Sears, 1961). It has also been used as a preservative for cosmetic products. In addition, it was reported that methylparaben is a chemical compound present in local anesthetic that can alter the function of the human nervous system (Nathan and Sears, 1961). Thus, we tested whether MB could affect the physiology of human neuronal cells, such as cell growth and proliferation, using a cultured human cancer cell line, SH-SY5Y. In the WST-1 assay, toxic effect of MB observed for neuronal cells was similar to kidney and colon cells, in which notable reduction in growth was observed at MB concentrations of >7.3 mM (0.1%, Figure 2C). We calculated the LC_50_ values of MB for HEK293, CACO2, and SH-Y5Y cells and summarized them in Table 1. These cytotoxicity results suggest that the overall, concentrations of MB > 7.3 mM (0.1%) appear inhibitory to the human cells as it limited their growth and proliferation.

### 3.3 MB has less cytotoxicity compared to ethyl and vinyl benzoate

**Figure 3:**
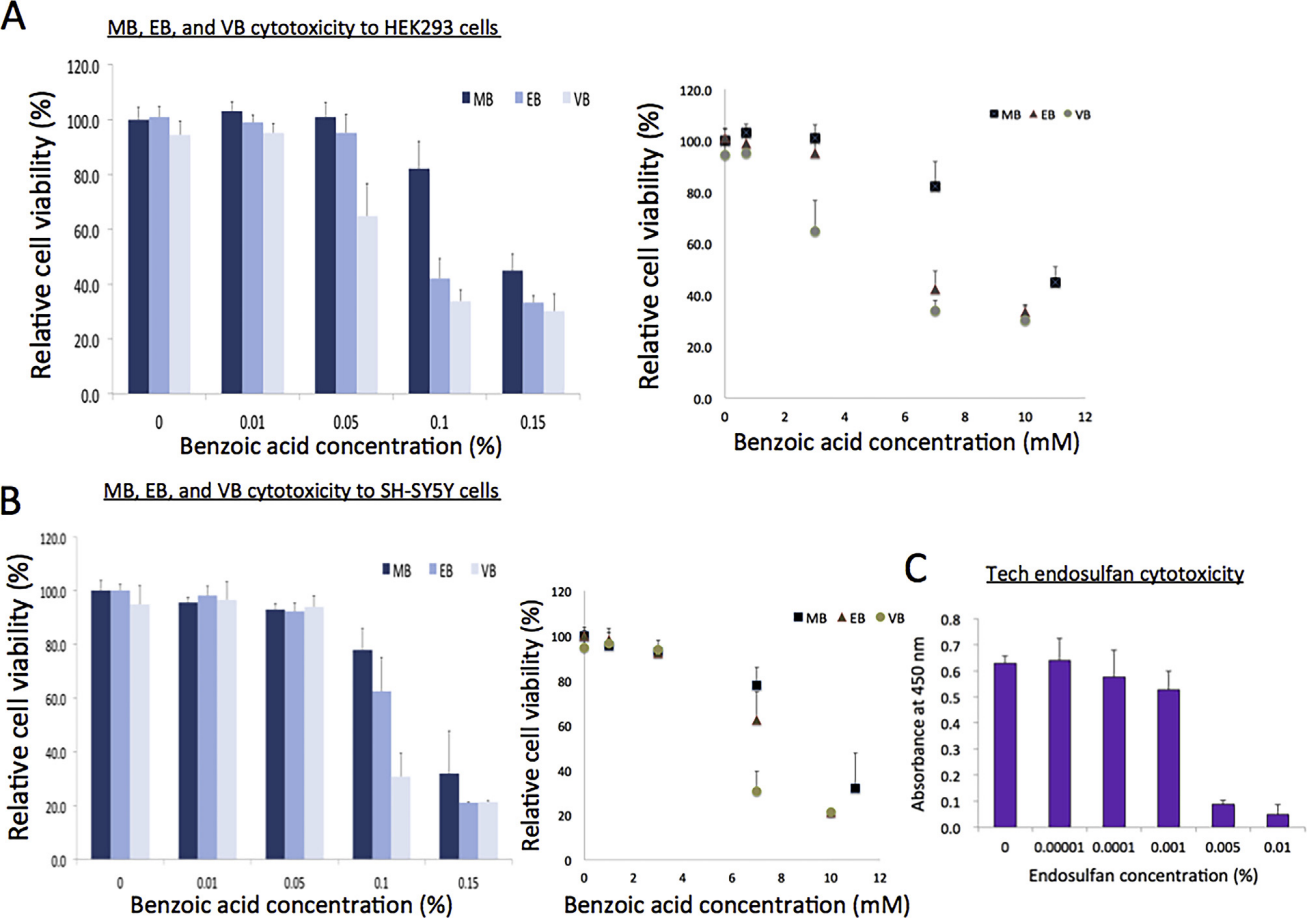
Cytotoxicity comparison with MB, EB, and VB against HEK293 and SH-SY5Y cells. (**A**) Results from the WST assays comparing MB, EB, and VB with HEK293 cells. Error bars, standard deviations (n = 10). Black bars, MB; dark grey, EB, light grey, VB. Acetone was used as a solvent. Left panel, the concentrations of benzoic acids presented in percentage (v/v); Right panel, these in molar concentrations. (**B**) Results from the WST assays comparing MB, EB, and VB with SH-SY5Y cells. Error bars, standard deviations (n = 10). Black bars, MB; dark grey, EB, light grey, VB. Left panel, the concentrations of benzoic acids presented in percentage (v/v); Right panel, these in molar concentrations. (**C**) Results from the WST assays with technical endosulfan and HEK293 cells. Error bars, standard deviation (n = 6).

We compared the cytotoxicity of MB with other commercially used benzoates, such as ethyl (EB, C_9_H_10_O_2_; MW, 150.17 g/mol) and vinyl benzoate (VB, C_9_H_8_O_2_, MW, 148.16 g/mol). EB is a natural compound, like MB, found in various fruits such as apples, bananas, and cherries and can be formed by the condensation of benzoate and ethanol (https://pubchem.ncbi.nlm.nih.gov/compound/Ethyl-benzoate). On the other hand, VB is a synthetic compound synthesized by the condensation of carboxyl group of benzoate acid with ethenol (https://pubchem.ncbi.nlm.nih.gov/compound/Vinyl-benzoate). EB is frequently used as an ingredient in fragrances and artificial flavors as listed in food chemical codex and US FDA, while VB is used as an industrial reagent (Labruere et al., 2010). The cytotoxicity assay, comparing these chemicals in HEK293 and SH-SY5Y cells, revealed that MB is least toxic, followed by EB, and then VB which is the most toxic to these tested cells (Figures 3A and B, Table 1). The LC_50_ value of VB is about half of MB, 5.4 mM and 6.1 mM (0.08% and 0.09%) for HEK293 and SH-SY5Y, respectively. Hence, the toxicity of these benzoates can be considered in proposing the maximum limit of dosage to be contained in commercial products. MB toxicity to human cells *in vitro* appeared to be modest. For example, it is much less toxic than technical endosulfan, a pesticide banned in many countries because of its hazardous effects (Figure 3C) (Weber et al., 2010).

**Figure 4:**
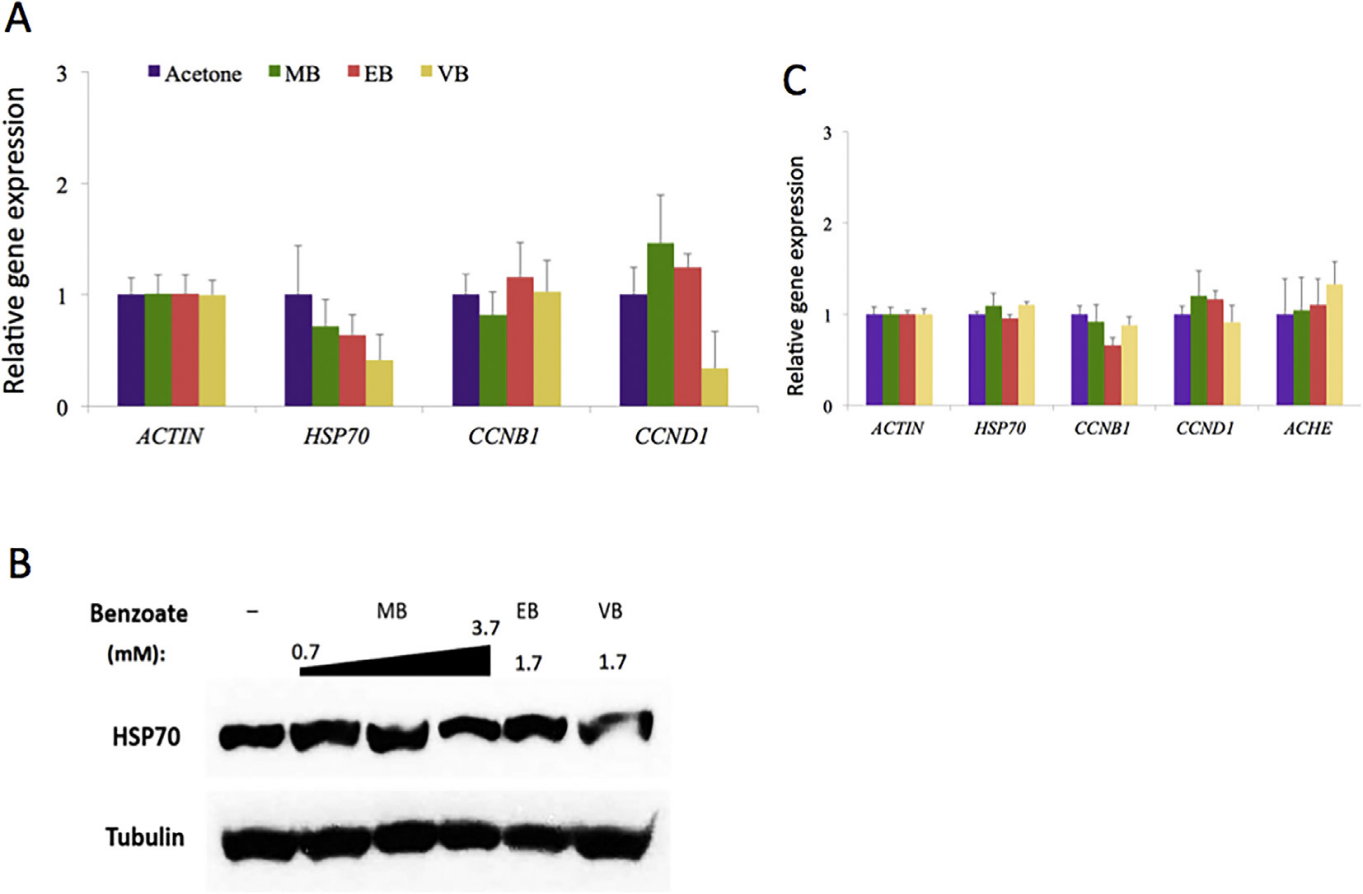
Gene expression of stress-induced and cell cycle regulators by MB, EB, and VB in HEK293 and SH-SY5Y cells. (**A**) qRT-PCR data showing the alteration of *HSP70* and *CCND1* (cyclin D1) genes. ACTIN was used as a reference gene. Error bars, standard deviations (n = 6). (**B**) Western blot results confirming the reduced protein level of HSP70 in the HEK293 cells treated with VB. α-Tubulin was used as a reference and loading control. Uncropped, non-adjusted images are shown in Figure S1. (**C**) qRT-PCR results. Error bars, standard deviations (n = 6).

Further, we prompted to assess whether the gene expression of cell cycle and stress regulators such as cyclins and HSP70 is affected by MB, EB, and VB. These chemicals were applied to the HEK293 or SH-SY5Y cells at the concentrations of 4.4 mM [0.06% MB, 0.05% (3.7 mM) for SH-SY5Y cells], 1.7 mM (0.025%, EB), and 1.7 mM (0.025%, VB), respectively, for 48 h before extracting the total RNAs from the cells. The concentrations were chosen based on the LC_50_ values, approximately a third of the values, that would not cause cell death but could influence the gene expression in the tested cells. mRNAs of *HSP70*, *CCNB1* (*cyclin B1*), and *CCND1* (*cyclin D1*) were quantified by real-time PCR with *ACTIN* as a reference gene. *Cyclin B1* is a checkpoint regulator for S/G_2_ transition, whereas *cyclin D1* is for G_1_ progression (Baldin et al., 1993; Hwang et al., 1995). It was found that *cyclin B1* mRNA expression was rarely differentiated in the cells treated with MB, EB, and VB compared with that in the control cells treated with the solvent (acetone) only (Figure 4A). However, we found a noticeable reduction of *cyclin D1* mRNA expression in VB-treated HEK293 cells (Figure 4A). The mRNA expression of *HSP70*, a stress-inducible gene (Huang et al., 2001), decreased in MB-, EB-, and VB-treated HEK293 cells, and VB-treated samples showing the least expression (Figure 4A). In addition, the protein level of HSP70 consistently decreased in VB-treated HEK293 cells (Figure 4B and S1). At the same concentrations, SH-SY5Y cells did not display notable differential expressions of these genes (Figure 4C). For SH-SY5Y cells, we also measured the mRNA expression of *AChE*, acetylcholinesterase gene (Dvir et al., 2010), because of the similar structure of these chemicals to methylparaben (Nathan and Sears, 1961) and of the fact that MB has been reported to have a repellent effect against some insect pests such as *Bemisia tabaci* (Gennadius) (Mostafiz et al., 2018). At the given concentrations, only VB-treated cells showed a mild increase in the level of *AChE* expression, whereas hardly any change was seen MB- and EB-treated cells (Figure 4C).

## 4 Conclusion

Hydrophobic chemicals are prone to be harmful after a certain range of concentrations (Bhaganna et al., 2010). Vitamin D is a good example. Although it is important and should not be deficient in human body, excess consumption of Vitamin D has been reported to cause risks and harms to humans (Marcinowska-Suchowierska et al., 2018). Although MB is naturally produced by plants and appears safe, it is a small hydrophobic substance that requires careful examination for toxicity and its safe dosage in humans should be established. We have found that MB can be lethal to HEK293, CACO2, and SH-SY5Y cells in high concentrations and have presented the LC_50_ values for these cells. Two other benzoates, EB and VB, were found to be more toxic than MB. These compounds, however appear to be less toxic compared to other pesticides such as technical endosulfan. Furthermore, we suggest that these benzoates when applied for human use should be at concentrations lower than the standard safe dosages determined through *in vitro* cell-basis and *in vivo* animal studies in the future.

Gene expression analyses have shown that VB reduces *cyclin D1* and *HSP7*0 mRNA and protein expression in HEK293 cells. Cyclin D1 is a critical protein for cell growth and proliferation (Baldin et al., 1993). The decrease of this protein is consistent with the inhibited cell growth and cytotoxicity exerted by VB. Reduced *HSP70* expression has been linked to compromised protein quality control and stress response as well as aging (Bobkova et al., 2015; Huang et al., 2001). Although further studies are required to understand the exact mechanism and consequences of HSP70 reduction, poor stress response may explain the toxicity displayed by VB to humans.

## Declarations

### Author contribution statement

TBC H. Bunch: Conceived and designed the experiments; Analyzed and interpreted the data; Contributed reagents, materials, analysis tools or data; Wrote the paper.

J. Park, H. Choe and M. Mostafiz: Performed the experiments; Analyzed and interpreted the data.

K. Lee: Analyzed and interpreted the data; Contributed reagents, materials, analysis tools or data.

J. Kim: Contributed reagents, materials, analysis tools or data.

### Funding statement

H. Bunch was supported by Kyungpook National University Research Fund, 2017.

### Competing interest statement

The authors declare no conflict of interest.

### Additional information

Supplementary content related to this article is availiable in Supplementary Figure 1.

## Appendix A Supplementary data

MB manuscript_Supplementary Information R4 MB manuscript_Supplementary Information R4
