# Supplementary material for: Evaluating cytotoxicity of methyl benzoate in vitro
Source: Heliyon. 2020 Feb 4;6(2):e03351. doi: 10.1016/j.heliyon.2020.e03351 (PMC7005452; doi:10.1016/j.heliyon.2020.e03351)
Supplement: MB manuscript_Supplementary Information R4 [file mmc1.pdf]

Evaluating cytotoxicity of methyl benzoate *in vitro*

Heeyoun Bunch[1]†, Jungeun Park[1], Hyeseung Choe[1], Md Munir Mostafiz[1], Jang-Eok Kim[1], Kyeong-Yeoll Lee[1,2]

correspondence to heeyounbunch@gmail.com

This PDF file includes:

Fig. S1

SUPPLEMENTARY FIGURES

Fig. S1.

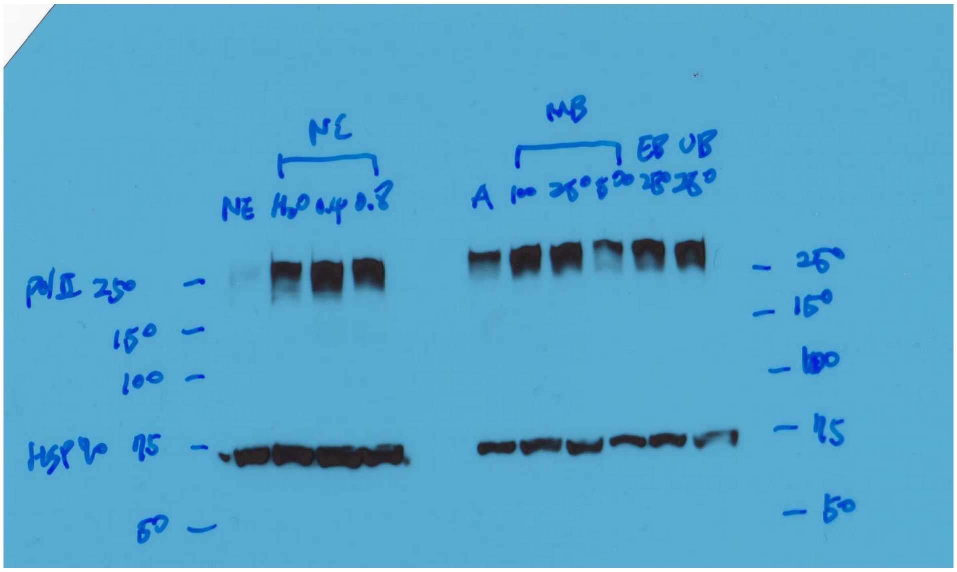

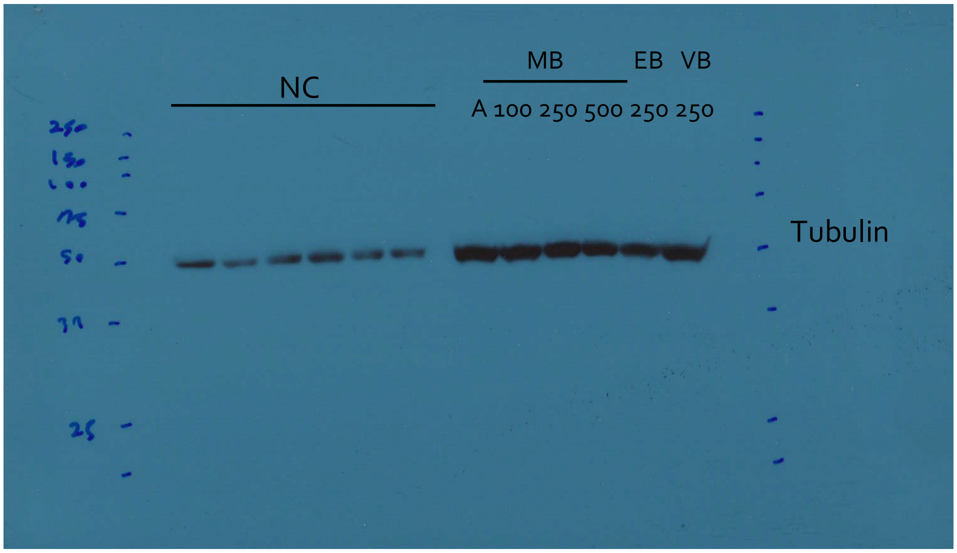

Supplementary Figure 1. Methyl benzoate (MB), ethyl benzoate (EB), and vinyl benzoate (VB) effect on HSP70 protein expression in HEK293 cells. Uncropped and unadjusted Western blot images are shown. HSP70 and Tubulin images shown above are the same exposure that was used in Figure 4B. Upper blot showing HSP70 protein expression: A, acetone only. MB concentrations of 0.7, 1.85, and 3.7 mM MB correspond to 100, 250, and 500 ppm as shown. EB and VB concentration 250 ppm corresponds to 1.7 mM. Bottom blot: Tubulin as an internal and loading control. In both blot images, NC labeled lanes are not related to the current study.
